# Structure-Dependent Spectroscopic Properties of Yb^3+^-Doped Phosphosilicate Glasses Modified by SiO_2_

**DOI:** 10.3390/ma10030241

**Published:** 2017-02-28

**Authors:** Ling Wang, Huidan Zeng, Bin Yang, Feng Ye, Jianding Chen, Guorong Chen, Andew T. Smith, Luyi Sun

**Affiliations:** 1 Key Laboratory for Ultrafine Materials of Ministry of Education, School of Materials Science and Engineering, East China University of Science and Technology, Shanghai 200237, China; y30140453@mail.ecust.edu.cn (L.W.); y45150059@mail.ecust.edu.cn (B.Y.); y30140482@mail.ecust.edu.cn (F.Y.); jiandingchen@ecust.edu.cn (J.C.); grchen@ecust.edu.cn (G.C.); 2 Department of Chemical and Biomolecular Engineering and Polymer Program, Institute of Materials Science, University of Connecticut, Storrs, CT 06269, USA; andrew.smith@uconn.edu (A.T.S.); luyi.sun@uconn.edu (L.S.)

**Keywords:** Yb^3+^-doped, phosphosilicate glasses, SiO_2_

## Abstract

Yb^3+^-doped phosphate glasses containing different amounts of SiO_2_ were successfully synthesized by the conventional melt-quenching method. The influence mechanism of SiO_2_ on the structural and spectroscopic properties was investigated systematically using the micro-Raman technique. It was worth noting that the glass with 26.7 mol % SiO_2_ possessed the longest fluorescence lifetime (1.51 ms), the highest gain coefficient (1.10 ms·pm^2^), the maximum Stark splitting manifold of ^2^F_7/2_ level (781 cm^−1^), and the largest scalar crystal-field N_J_ and Yb^3+^ asymmetry degree. Micro-Raman spectra revealed that introducing SiO_2_ promoted the formation of P=O linkages, but broke the P=O linkages when the SiO_2_ content was greater than 26.7 mol %. Based on the previous ^29^Si MAS NMR experimental results, these findings further demonstrated that the formation of [SiO_6_] may significantly affect the formation of P=O linkages, and thus influences the spectroscopic properties of the glass. These results indicate that phosphosilicate glasses may have potential applications as a Yb^3+^-doped gain medium for solid-state lasers and optical fiber amplifiers.

## 1. Introduction

Yb^3+^-doped laser materials operating at wavelengths around 1 μm have been intensively investigated for a wide variety of applications, such as high-power and short-pulse lasers, material processing, and optical telecommunications [1,2,3,4]. Yb^3+^ ions are regarded as the main dopant for the applications because of their simple energy-level scheme, which prevents excited-state absorption and multi-phonon non-radiative decay, and obviates the possibility of concentration quenching through cross-relaxation [5]. Since the first glass laser was obtained in 1961 by Snitzer [6], Yb^3+^-doped glasses have been well established as solid-state lasers and optical fiber amplifiers for optical telecommunications. Recently, for high-power glass-based laser systems, phosphate glasses have been used as a matrix for Yb^3+^ ions because of their high rare-earth solubility, high gain coefficient and superior spectroscopic properties [7,8,9]. However, the predominant disadvantages of phosphate glasses are their chemical durability and thermo-mechanical limitations. Therefore, optimizing the glass compositions with significantly improved thermo-mechanical properties is required.

Silicate glasses exhibit excellent chemical durability, thermo-mechanical properties and optical properties. Recent studies have shown that the mechanical properties of phosphate glasses can be efficiently improved by doping with SiO_2_ [10,11,12]. Chen Wei et al. [11] suggested that the introduction of SiO_2_ into phosphate glasses can strengthen the thermo-mechanical properties of the glass without severely degrading the spectroscopic properties. Zhang Liyan et al. [12] reported that the spectroscopic properties of 60P_2_O_5_-7.5Al_2_O_3_-15K_2_O-17.5BaO glass can be improved by the addition of SiO_2_. Moreover, the Stark splitting of Yb^3+^-doped phosphate glasses is enlarged through the introduction of SiO_2_, which allows the glass to achieve the laser output successfully. The glass structure and the local coordination of rare-earth ions can be effectively modulated by doping SiO_2_ into phosphate glasses which critically influences the spectroscopic properties of the glass. Zeng Huidan et al. [13] reported that both the luminous intensity and luminous decay time of the glass appeared to have positive correlations with the amount of bridging oxygen of the glass matrix through using X-ray photoelectron spectroscopy (XPS). Hu Lili et al. [14] reported the mechanism for the decrease in Yb^3+^ absorption and emission intensity caused by P^5+^ doping. They found that Yb^3+^ coordinated to the P–O site in glass with a molar ratio of P^5+^/Al^3+^ ≤ 1, and coordinated to the P=O site in glass with a molar ratio of P^5+^/Al^3+^ > 1.

In this study, Yb^3+^-doped phosphate glasses in the system BaO-P_2_O_5_ were modified by the addition of SiO_2_. The scalar crystal-field N_J_ and Yb^3+^ asymmetry degrees were calculated from the Stark splitting levels, which were derived from Lorentz fitting based on the absorption and emission spectra. Furthermore, the influence mechanism of SiO_2_ on the structural and spectroscopic properties was investigated systematically using the micro-Raman technique and previous ^29^Si MAS NMR experimental results. The results may have certain implications for the realization of a new generation of high-power solid-state lasers for optical telecommunications applications.

## 2. Experimental

Yb^3+^-doped silicophosphate glasses with compositions (in mol %) 20BaO-(80-*x*)P_2_O_5_-*x*SiO_2_-1Yb_2_O_3_ (*x* = 9, 16, 26.7, 32, and 40 mol %, respectively) were prepared by conventional melt-quenching technique. High purity BaCO_3_, NH_4_H_2_PO_4_, SiO_2_ from Sinopharm Chemical Reagent Company (Ning Bo, China), and 99.99% Yb_2_O_3_ from Macklin were used as starting materials for preparation of the glasses. About 20 g of raw materials were thoroughly crushed in an agate mortar and the homogeneous mixture was transferred into a corundum crucible, which was preheated at 350 °C for 30 min before being fully melted at 1350–1400 °C for 45 min under continuous stirring. Molten glass was air quenched by casting it onto a preheated brass mold to form bulk glasses and annealed at 430–480 °C for 5 h to reduce the thermal stress and strains. Then the furnace was switched off and the glass was allowed to cool down to room temperature at a cooling rate of about 3 K·min^−1^. A slab of 10 mm × 10 mm × 2 mm sample was cut from the specimens and both sides were optically polished for the spectroscopic measurements.

The UV-VIS-NIR absorption spectra of BaO-P_2_O_5_-SiO_2_ glasses were measured using a Varian CARY 500 spectrophotometer (Varian Inc., Palo Alto, CA, USA) in the scanning range of 800–1100 nm. With 915 nm laser diode pump, the emission spectra and lifetimes were measured by a high resolution spectrofluorometer FLSP920 cooled with liquid helium (Edinburgh Instruments Ltd., Livingston, UK). A scanning step of 1 nm was used to measure both absorption and emission spectra. The structural information on glass samples was obtained by micro-Raman spectrometer (INVIA, Renishaw, Gloucestershire, UK) with an Ar^+^-ion laser (514.5 nm) as the irradiation source. Baseline correction was performed using the Wire software program from Renishaw. All the measurements were performed at room temperature.

## 3. Results and Discussion

**Figure 1:**
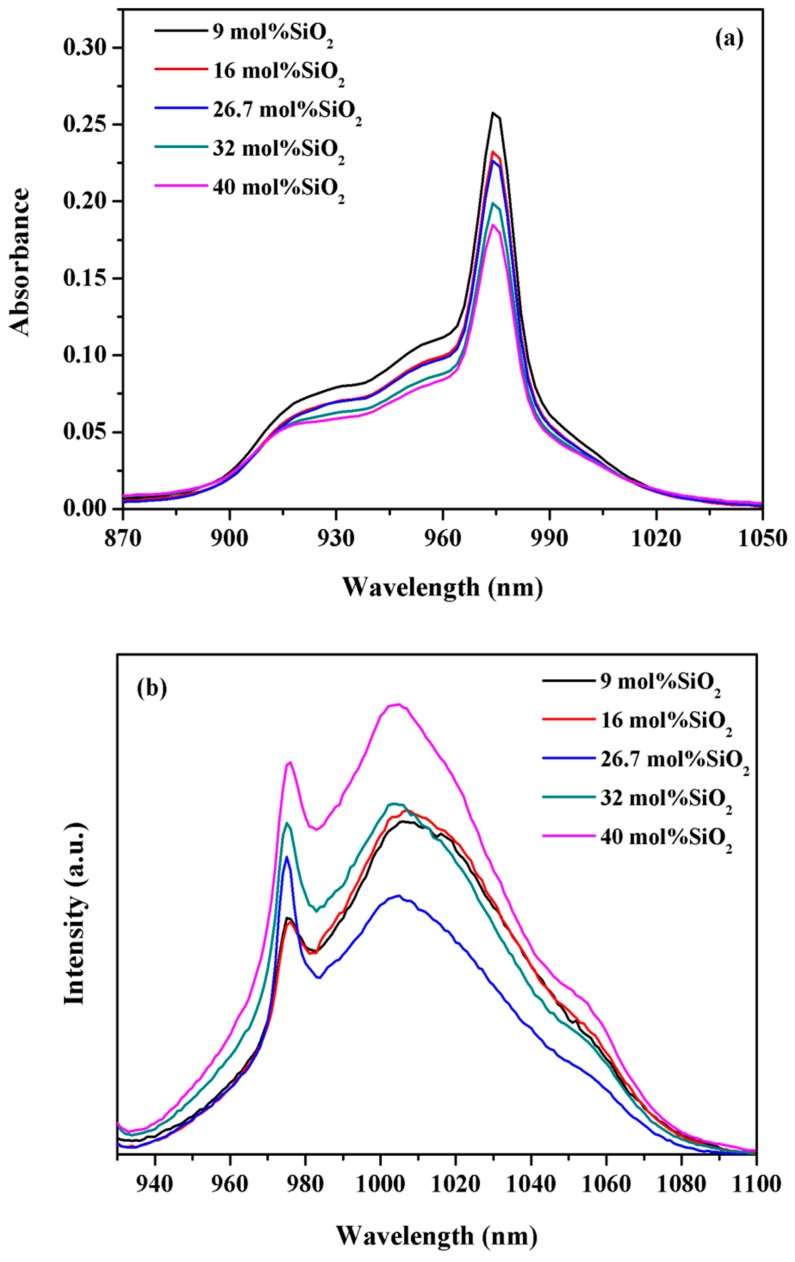
Absorption (**a**) and emission (**b**) spectra of 20BaO-(80-*x*)P_2_O_5_-*x*SiO_2_-1Yb_2_O_3_ (*x* = 9, 16, 26.7, 32, and 40 mol %, respectively) glasses.

The absorption and emission spectra of 20BaO-(80-*x*)P_2_O_5_-*x*SiO_2_-1Yb_2_O_3_ (*x* = 9, 16, 26.7, 32, and 40 mol %, respectively) glasses are plotted in Figure 1. As shown in this figure, the absorption band of the ^2^F_5/2_→^2^F_7/2_ transition was at 975 nm which corresponds to the transition between the lowest level of the ^2^F_5/2_ and ^2^F_7/2_ manifolds. The absorption intensity of glass samples decreased with the increasing SiO_2_ content. Under excitation with 915 nm LDs (Laser Diodes), NIR emission peaks at around 975 and 1005 nm were observed. The SiO_2_ addition resulted in an increase in the emission intensity at around 975 nm. One broad emission band with the peak centered at 1005 nm was obtained upon excitation by 915 nm. The emission intensity decreased with the increased concentration of SiO_2_ up to 26.7 mol %, and then increased as shown in Figure 1b. The variation trend of the luminescent intensity was different from the trend of the absorption intensity, which means other factors must exist that are able to affect the luminescent intensity.

**Figure 2:**
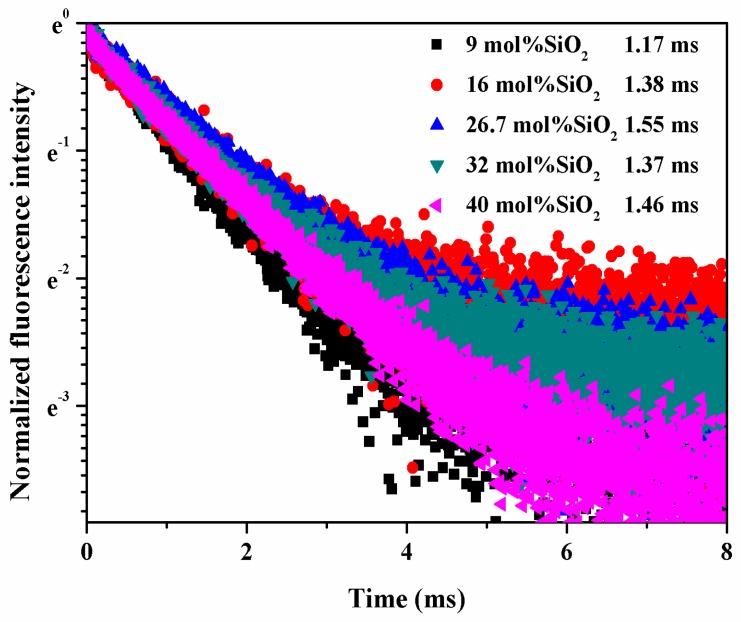
The fluorescence decay curve of Yb^3+^-doped 20BaO-(80-*x*)P_2_O_5_-*x*SiO_2_-1Yb_2_O_3_ (*x* = 9, 16, 26.7, 32, and 40 mol %, respectively) glasses.

The lifetime of luminescent ions is a critical parameter for broadband optical amplifiers. The compositional dependences of emission lifetimes are shown in Figure 2. Apparently, the lifetime increases monotonically with the increase of the SiO_2_ content up to 26.7 mol %, and then decreases slightly with further increasing the content of SiO_2_. Besides the lifetime, the absorption and stimulated emission cross-sections are also an important factor for solid-state lasers and broadband optical amplifiers. The absorption and emission cross-sections have been calculated by the reciprocity method [15,16]; the absolute value of cross-sections and accurate spectra information can be obtained in Table 1. As shown in Table 1, the absorption and emission cross-sections of 20BaO-(80-*x*)P_2_O_5_-*x*SiO_2_-1Yb_2_O_3_ (*x* = 9, 16, 26.7, 32, and 40 mol %, respectively) glass samples decreased with the increasing SiO_2_ concentration. The magnitude of the absorption (emission) cross-section at 975 nm for all the studied Yb^3+^-doped glass was found to be in the range of 0.62–1.09 × 10^−20^ (0.83–1.46 × 10^−20^ cm^2^), which is much higher than those of the commercial Kigre QX/Yb: 0.50 × 10^−20^ (1.06 × 10^−20^ cm^2^) laser glass [17]. The product (σ_em_ × τ_exp_) of the stimulated emission cross-section and the lifetime is a significant parameter to depict laser materials for the laser threshold is inversely proportional to σ_em_ × τ_exp_. The σ_em_ × τ_exp_ values of the Yb^3+^-doped phosphosilicate glass are shown in Table 1. All the σ_em_ × τ_exp_ values of this work were about 1 × 10^-23^ cm^2^s, which indicates that these glasses could be a potential material for high-power solid-state lasers and broadband optical amplifiers.

**Figure 3:**
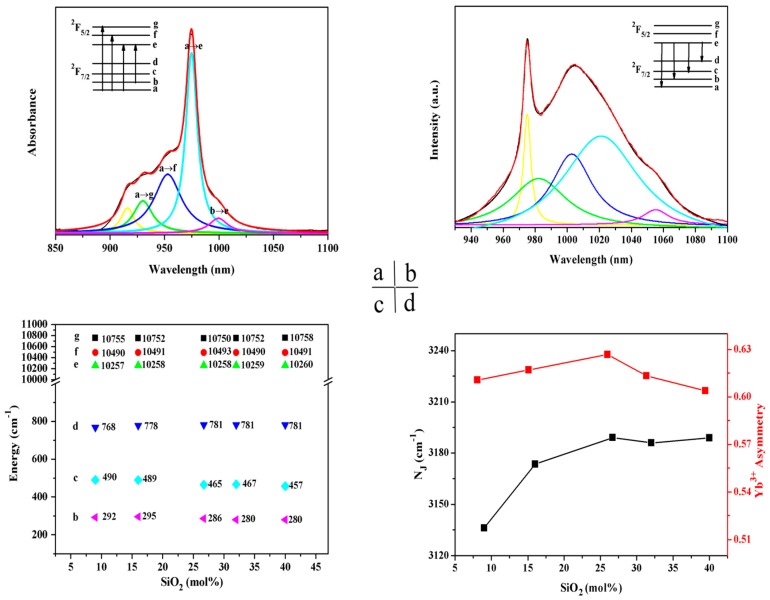
Lorentz peak analysis for absorption (**a**) and emission (**b**) spectra of 20BaO-53.3P_2_O_5_-26.7SiO_2_-1Yb_2_O_3_ glass (the black lines are the original spectra, while the red lines offer the fitting lines composed of the corresponding multi-fitting peaks); (**c**) Stark level energies of ^2^F_7/2_ and ^2^F_5/2_ manifolds in 20BaO-(80-*x*)P_2_O_5_-*x*SiO_2_-1Yb_2_O_3_ (*x* = 9, 16, 26.7, 32, and 40 mol %, respectively) glasses obtained from the Lorentz fitting to the absorption and emission spectra; (**d**) Scalar crystal-field parameters N_J_ and Yb^3+^ asymmetry degree in 20BaO-(80-*x*)P_2_O_5_-*x*SiO_2_-1Yb_2_O_3_ (*x* = 9, 16, 26.7, 32, and 40 mol %, respectively) glasses.

Recently, many research studies have been published on NIR luminescence in Yb^3+^-doped glasses; however, the origin of this phenomenon has not been identified. The relation between the glass structure and the spectroscopic properties of Yb^3+^-doped glass is revealed through the evaluation of the scalar crystal-field N_J_ and Yb^3+^ asymmetry degree. According to References [18,19,20], the scalar crystal-field N_J_ and Yb^3+^ asymmetry degree can be calculated from the Stark splitting levels, which can be derived from Lorentz fitting based on the absorption and emission spectra. As shown in Figure 3, the maximum Stark splitting manifold of the ^2^F_7/2_ level (781 cm^−1^) and the scalar crystal-field N_J_ and Yb^3+^ asymmetry degree are observed when the SiO_2_ concentration is 26.7 mol %.

**Figure 4:**
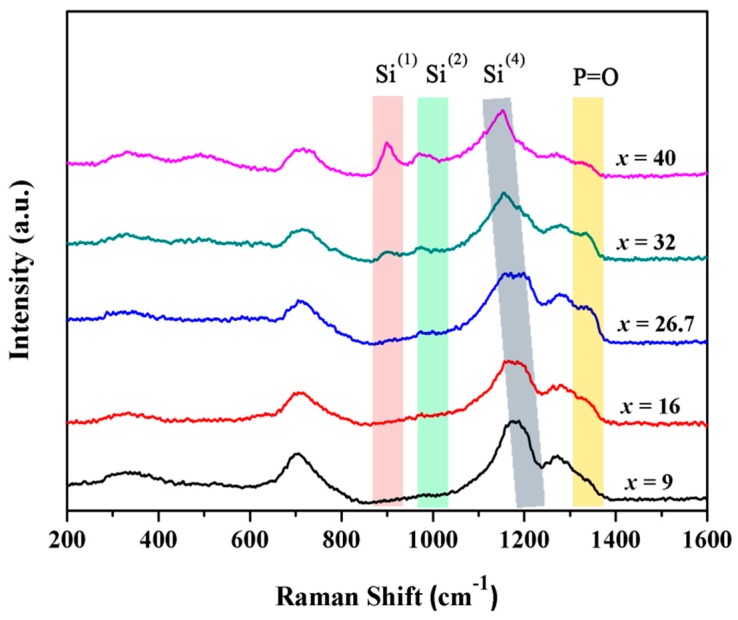
Micro-Raman spectra of 20BaO-(80-*x*)P_2_O_5_-*x*SiO_2_-1Yb_2_O_3_ (*x* = 9, 16, 26.7, 32, and 40 mol %, respectively) glasses.

As is known, introducing SiO_2_ into phosphate glass can effectively modulate the structure and thus lead to a change in the Yb^3+^ local field. Therefore, to further elucidate the role of SiO_2_ in phosphate glass, the detailed structural information of the glass by using the micro-Raman technique was obtained. In Figure 4, micro-Raman spectra are shown as a function of an increasing SiO_2_ content in the range of 200–1600 cm^−1^. The broad bands of the Si^(*n*)^ units (Si^(*n*)^ represents the [SiO_4_] tetrahedral unit and n is the amount of bridging oxygen per tetrahedral) with *n* = 4, 3, 2, 1 and 0, which are centered at around 1200, 1100, 950, 900, and 850 cm^−1^, respectively [21]. The spectra of low-SiO_2_ glass show four major features centered near 700, 1155, 1277, and 1330 cm^−1^, respectively. With an increasing content of SiO_2_, several new peaks appear at 500, 900, and 970 cm^−1^. The bands near 900, 970, 1155 cm^−1^ are assigned to Si^(1)^, Si^(2)^, and Si^(4)^, respectively. As shown in Figure 4, the band near 1155 cm^−1^ contributing to the stretching vibration mode in Si^(4)^ becomes wider and moves towards a lower wave number. This may be due to the formation of [SiO_6_] which broadens the peak near 1155 cm^−1^ [22,23,24]. The band near 1330 nm is derived from P=O stretching vibration [25,26]. As the content of SiO_2_ is increased, the intensity of the Raman peak increases until 26.7 mol % SiO_2_ and then it decreases. This structural change indicates that the introduction of SiO_2_ can promote the formation of P=O linkages, but it can also break the P=O linkages when the SiO_2_ content is greater than 26.7 mol %. P=O linkages arouse a remarkable adjustment on the distorted structure and thus result in a dramatic change in the Yb^3+^ local structure. As shown in Figure 3d, the variation trend of the asymmetry degree and N_J_ is similar to that of the P=O linkage. According to previous work [27], ^29^Si MAS NMR spectra of 20BaO-(80-*x*)P_2_O_5_-*x*SiO_2_ (*x* = 9, 16, 26.7, 32, and 40 mol %, respectively) glass samples indicated that [SiO_6_] existed in these phosphosilicate glasses, and the peaks of [SiO_6_] significantly decreased when the SiO_2_ content was greater than 26.7 mol %. Based on the previous ^29^Si MAS NMR and micro-Raman experimental results, these findings further demonstrate that the presence of [SiO_6_] may significantly affect the formation of P=O, and thus improve the spectroscopic properties of phosphate glasses.

## 4. Conclusions

The influence mechanism of SiO_2_ on the structural and spectroscopic properties of phosphate glasses prepared by the conventional melt-quenching method was systematically investigated using the micro-Raman technique and previous ^29^Si MAS NMR analysis. A significant change occurs in the variation trends of fluorescence lifetimes and the scalar crystal-field N_J_, and Yb^3+^ asymmetry degree when the SiO_2_ content is greater than 26.7 mol %. It is worth noting that the glass with 26.7 mol % SiO_2_ possess the longest fluorescence lifetime (1.51 ms), the highest gain coefficient (1.10 ms·pm^2^), the maximum Stark splitting manifold of ^2^F_7/2_ level (781 cm^−1^), and the greatest N_J_ and Yb^3+^ asymmetry degree. Micro-Raman spectra indicate that the formation of P=O linkages in the glass is responsible for this abnormal variation. With the increase in the SiO_2_ concentration, the intensity of the P=O linkages increases, and then slightly decreases when the SiO_2_ content is greater than 26.7 mol %. This variation trend is consistent with the N_J_ and Yb^3+^ asymmetry degree. Additionally, based on the previous ^29^Si MAS NMR experimental results, [SiO_6_] units existing in these phosphosilicate glasses may significantly affect the formation of P=O, and thus influence the spectroscopic properties of the glasses. It can be realized that these phosphosilicate glasses could be materials possessing the potential to be developed as a Yb^3+^-doped gain medium for high-power solid-state lasers and broadband optical amplifiers.

## Figures and Tables

**Table 1:** Spectral parameters for 20BaO-(80-*x*)P_2_O_5_-*x*SiO_2_-1Yb_2_O_3_ (BPS*x*) (*x* = 9, 16, 26.7, 32, and 40 mol %, respectively) glass samples.

Glass	σ_abs_ (975 nm) (10^−20^ cm^2^)	σ_em_(975 nm) (10^−20^ cm^2^)	σ_abs_ (1005 nm) (10^−20^ cm^2^)	σ_em_ (1005 nm) (10^−20^ cm^2^)	τ_exp_ (ms)	σ_em_ × τ_exp_ (10^−20^ cm^2^·ms)
BPS9	1.09	1.46	0.15	0.86	1.17	1.01
BPS16	0.98	1.30	0.13	0.76	1.38	1.04
BPS26.7	0.85	1.13	0.12	0.71	1.55	1.10
BPS32	0.80	1.07	0.11	0.66	1.37	0.91
BPS40	0.62	0.83	0.11	0.62	1.46	0.90
